# Role of *GUCA1C* in Primary Congenital Glaucoma and in the Retina: Functional Evaluation in Zebrafish

**DOI:** 10.3390/genes11050550

**Published:** 2020-05-14

**Authors:** Samuel Morales-Cámara, Susana Alexandre-Moreno, Juan-Manuel Bonet-Fernández, Raquel Atienzar-Aroca, José-Daniel Aroca-Aguilar, Jesús-José Ferre-Fernández, Carmen-Dora Méndez, Laura Morales, Laura Fernández-Sánchez, Nicolas Cuenca, Miguel Coca-Prados, José-María Martínez-de-la-Casa, Julián Garcia-Feijoo, Julio Escribano

**Affiliations:** 1 Área de Genética, Facultad de Medicina de Albacete/Instituto de Investigación en Discapacidades Neurológicas (IDINE), Universidad de Castilla-La Mancha, 02006 Albacete, Spain; samuelmoralescamara@gmail.com (S.M.-C.); Susana.Alexandre@uclm.es (S.A.-M.); JuanM.Bonet@uclm.es (J.-M.B.-F.); Raquel.Atienzar@uclm.es (R.A.-A.); josedaniel.aroca@uclm.es (J.-D.A.-A.); ferrejesus@hotmail.com (J.-J.F.-F.); 2 Cooperative Research Network on Age-Related Ocular Pathology, Visual and Life Quality (OFTARED), Instituto de Salud Carlos III, 28029 Madrid, Spain; cmendez@iies.es (C.-D.M.); lauramoralesfernandez@gmail.com (L.M.); cuenca@ua.es (N.C.); jmmartinezcasa@gmail.com (J.-M.M.-d.-l.-C.); jgarciafeijoo@hotmail.com (J.G.-F.); 3 Servicio de Oftalmología, Hospital San Carlos, 28040 Madrid, Spain; 4 Instituto de Investigación Sanitaria del Hospital Clínico San Carlos, 28040 Madrid, Spain; 5 Department of Optics, Pharmacology and Anatomy, University of Alicante, 03690 Alicante, Spain; laura.fs@gcloud.ua.es; 6 Department of Physiology, Genetics and Microbiology, University of Alicante, 03690 Alicante, Spain; 7 Department of Ophthalmology and Visual Science, Yale University School of Medicine, New Haven, CT 06510, USA; miguel.coca-prados@yale.edu

**Keywords:** primary congenital glaucoma, exome sequencing, GUCA1C, GCAP3, zebrafish, CRISPR/Cas9

## Abstract

Primary congenital glaucoma (PCG) is a heterogeneous, inherited, and severe optical neuropathy caused by apoptotic degeneration of the retinal ganglion cell layer. Whole-exome sequencing analysis of one PCG family identified two affected siblings who carried a low-frequency homozygous nonsense *GUCA1C* variant (c.52G > T/p.Glu18Ter/rs143174402). This gene encodes GCAP3, a member of the guanylate cyclase activating protein family, involved in phototransduction and with a potential role in intraocular pressure regulation. Segregation analysis supported the notion that the variant was coinherited with the disease in an autosomal recessive fashion. GCAP3 was detected immunohistochemically in the adult human ocular ciliary epithelium and retina. To evaluate the ocular effect of *GUCA1C* loss-of-function, a *guca1c* knockout zebrafish line was generated by CRISPR/Cas9 genome editing. Immunohistochemistry demonstrated the presence of GCAP3 in the non-pigmented ciliary epithelium and retina of adult wild-type fishes. Knockout animals presented up-regulation of the glial fibrillary acidic protein in Müller cells and evidence of retinal ganglion cell apoptosis, indicating the existence of gliosis and glaucoma-like retinal damage. In summary, our data provide evidence for the role of *GUCA1C* as a candidate gene in PCG and offer new insights into the function of this gene in the ocular anterior segment and the retina.

## 1. Introduction

Glaucoma is a heterogeneous group of progressive and irreversible optic neuropathies stemming from the apoptotic death of retinal ganglion cells. It results in characteristic visual field loss. Common glaucoma types have an adult onset, though infrequent early onset forms of glaucoma are also a significant cause of visual disability. Glaucoma that occurs before three years of age and associates with isolated trabeculodysgenesis is called primary congenital glaucoma (PCG) [1]. Arrested maturation of tissues derived from cranial neural crest cells is believed to underlie this disease. This alteration results in increased aqueous humor (AH) outflow resistance, elevated intraocular pressure (IOP), and optic nerve degeneration and usually manifests in the form of the classic triad of tearing, photophobia and corneal clouding. PCG affects one in 10,000–20,000 live births in Western countries [2], with an increased incidence in consanguineous populations [3,4]. Although PCG has a poorly understood pathogenesis, it has a strong genetic component and is frequently inherited as an autosomal-recessive trait with incomplete penetrance (ranging from 40% to 100% [5]) and variable expressivity. The first identified and most prevalent cause of this type of inheritance is loss-of-function (LoF) of the *CYP1B1* (*CYTOCHROME P450*, *SUBFAMILY I*, *POLYPEPTIDE 1*, MIM# 601771) gene [5], which is present in 18–48% of non-consanguineous European patients [6,7]. Our previous studies showed that approximately one-third of Spanish PCG patients carry *CYP1B1* pathogenic genotypes [8,9]. Several other disrupted genes have also been identified in this disease, illustrating the genetic heterogeneity of PCG. LoF of the *LATENT TRANSFORMING GROWTH FACTOR-β-BINDING PROTEIN 2* (*LTBP2*, MIM# 602091) gene results in occurrence of the disease in a reduced percentage of patients [10,11,12]. Rare variants in the angiopoietin receptor *TEK* (*TEK*, MIM# 600221) likely underlie dominant PCG with variable expressivity in some patients [13]. Additionally, rare hypermorphic *G-PATCH DOMAIN-CONTAINING PROTEIN 3* (*GPATCH3*, MIM# 617486) variants have been reported in some cases [14]. The presence of disease-causing variants in other genes such as *MYOCILIN* (*MYOC*, MIM# 601652) [15,16] and *FORKHEAD BOX C1* (*FOXC1*, MIM# 601090) [17,18] and *CPAMD8* [19,20] have also been described in a few patients.

Herein, we extend our previous family-based whole-exome sequencing (WES) study to identify PCG-causing variants in 26 non-related probands. We identified the presence of one low-frequency homozygous nonsense *GUCA1C* variant in two siblings, inherited in a recessive fashion. The role of *GUCA1C* in the disease was also evaluated by expression analyses in ocular human tissues and functional studies in zebrafish. Our data offer novel insights into the genetics of this disease and the functional role of *GUCA1C* in the ocular anterior segment and the retina.

## 2. Materials and Methods 

### 2.1. Subjects

The family reported in this study belong to a cohort of 26 PCG previously analyzed by whole-exome sequencing [14]. Glaucoma specialists carried out the clinical examination of the patient and PCG diagnosis was performed as previously described [9]. The human study and informed consent procedures were approved by the Ethics Committee for Human Research of the Hospital Clínico San Carlos (approval number 13/388-E). The research followed the tenets of the Declaration of Helsinki. Informed written consents were obtained prior to participants’ inclusion in the study.

### 2.2. Human Tissue Samples

A human eye from a 45-year-old Caucasian female donor (cadaver) with no reported ocular pathology was obtained within 24 h after enucleation from the USA National Disease Research Interchange. The eye was microdissected from the posterior pole and the vitreous and aqueous humor were collected. Then, the eye was fixed with 4% paraformaldehyde in 0.1 phosphate buffer (pH 7.2) and embedded in paraffin as previously reported [21]. Histological microtome sections (10 μm) were deparaffinized for immunohistochemical analysis.

### 2.3. Animals

Wild-type AB zebrafish (*Danio rerio*) were maintained at 28 °C with a 14 h on/10 h off light cycle and were fed a standard diet according to established protocols [22]. Zebrafish embryos were raised at 28 °C in E3 medium (5 mM NaCl; 0.17 mM KCl; 0.33 mM CaCl_2_; 0.33 mM MgSO_4_, and 0.0001% methylene blue, pH 7.2). All animal husbandry and experiments were approved and conducted in accordance with the guidelines set forth by the Institutional Animal Research Committee of the University of Castilla-La Mancha (approval number PR-2015-04-10).

### 2.4. Next Generation Sequencing (NGS)

Genomic DNA was extracted from the subjects’ peripheral blood, using the *QIAamp DNA Blood Mini Kit* (Quiagen, Hilden, Germany) and processed for NGS as previously described [14]. Candidate disease-causing variants were identified through the application of a multistep filtering approach. Initially, common variants, defined as those with a minor allele frequency higher than 1% in the Exome Aggregation Consortium (ExAC) (http://exac.broadinstitute.org/) or gnomAD (https://gnomad.broadinstitute.org/) databases and with a genotype quality lower than 50 reads were filtered out. Next, LoF variants (nonsense, indels producing a frameshift and variants affecting canonical splicing sites) were selected. Finally, to identify potential recessive genotypes, we selected variants in compound heterozygosis or homozygosis. The candidate variant identified by NGS was confirmed and segregated in the family by Sanger sequencing.

### 2.5. Quantitative Reverse Transcription PCR (qRT-PCR)

RNA was isolated from pools of 50 zebrafish larvae (6 dpf) using the RNeasy Minikit (Qiagen #74104) and treated with RNase-free DNase I according to the manufacturer’s instructions. Purified RNA was used for cDNA synthesis using RevertAid First Strand cDNA Synthesis Kits (Thermo Scientific #K1622). The expression of *guca1c* mRNA relative to *ef1α* mRNA was determined by the 2^−ΔΔCt^ method [23] using the following primer pairs, respectively: guca1cE2FW, 5′-ACGGCAAGATCGACAGAGATGAAATG-3′/guca1cE2Rv, 5′-CCTCTCATAGATCAGGCTCACG-3′ and ef1αFw, 5′-CTGGAGGCCAGCTCAAACAT-3′/ef1αRv, 5′-ATCAAGAAGAGTAGTACCG CTAGCATTAC-3′. PCR analysis was carried out with cDNA as a template in a reaction volume of 10 μL containing 5 μL of Power SYBR Green PCR Master Mix (Thermo Fisher Scientific, Waltham, MA, USA) and 200 nM of each primer. Thermocycling included an initial denaturation step at 95 °C for 10 min, followed by 40 cycles consisting of 15 s denaturation at 95 °C for 60 s and a combined annealing and extension step at 60° C for 40 s. DNA amplifications were carried out in an ABI PRISM 7500 Fast real-time PCR system (Life Technologies, Foster City, CA, USA). Template cDNA was omitted in the qRT-PCR negative control. qRT-PCR results from at least three independent experiments carried out in triplicate were used for calculation of mean expression values in each sample. 

### 2.6. Western Blotting and Antibodies

For western blot analysis of GCAP3 in *guca1c* KO zebrafish, eight embryos (6 dpf) per *guca1c* genotype (mutant homozygote, heterozygote and wild-type) were lysed, and 60 μg of total protein were fractionated by SDS-PAGE using the Mini-PROTEAN III Gel Electrophoresis System (BIORAD, Hercules, CA, USA). Then, proteins were transferred onto Hybond ECL nitrocellulose membranes (Amersham, Arlington Heights, IL, USA) as previously described [24]. GCAP3 was detected using a commercial rabbit polyclonal primary antibody (anti-human GUCA1C, HPA041597, Sigma-Aldrich, St. Louis, MO, USA) (1:50). Horseradish peroxidase-conjugated anti-rabbit IgG (#1858415, Pierce) was uses as a secondary antibody (1:1000). Chemiluminescence detection was performed as previously described [24].

### 2.7. Light Microscopy

Zebrafish tissue samples employed for light microscopy were immediately fixed in 2.5% glutaraldehyde/4% paraformaldehyde in 0.1 M Millonig’s phosphate buffer (PBM, pH 7.4), for 4 h at 4 °C, washed in PBM and dehydrated in ascending grades of acetone (30–100%) and embedded in araldite resin. Semi-thin sections (0.5 μm) were stained with toluidine blue and analyzed by light microscopy.

### 2.8. Fluorescence Immunohistochemistry (FIHC)

Paraffin-embedded human eye sections (10 μm) were obtained with a RM 2135 BioCut Rotary Microtome (Leica Ltd., Wetzlar, Germany). Zebrafish embryos (96 hpf) and heads of both adult (6 months) wild-type and knockout (KO) *guca1c* zebrafish were fixed in 4% paraformaldehyde overnight and cryoprotected in 30% sucrose/0.1 M DPBS for two days at 4 °C. Thereafter, the samples were embedded and oriented in 10% porcine gelatin with 15% sucrose and stored at −80 °C. Cryosections (10–14 μm) were obtained in a Leica CM3050 S cryostat and stored at −80 °C. Tissue sections were blocked in blocking solution [10% fetal bovine serum (FBS), 1% DMSO and 1% Triton X-100 in DPBS] at room temperature for 1 h. Thereafter, sections were incubated with primary antibodies at optimal dilution [rabbit anti-GCAP3 (1:200) (HPA041597, Sigma) or mouse anti-GFAP (1:50) (sc-33673, Santa Cruz Biotechnology, Santa. Cruz, CA, USA)] overnight at 4 °C. Then, after wash, sections were incubated with the corresponding secondary antibody: Cy2 donkey anti-rabbit (1:1000) (Jackson ImmunoResearch, West Grove, PA, USA) or Cy2 donkey anti-mouse (1:1000) (Jackson ImmunoResearch). All tissue sections were counterstained with DAPI (D8417, Sigma-Aldrich) for nuclear staining, mounted in Fluoroshield Medium (F6182, Sigma-Aldrich) and visualized using a LSM710 confocal microscope (Carl Zeiss, Jena, Germany). Fluorescence emitted by DAPI, the Cy2-conjugated antibody and embryo autofluorescence was registered at the following wavelengths, respectively: 411–464 nm, 490–518 nm and 553–677 nm. Negative controls were made omitting the primary antibodies.

Apoptotic cell death was evaluated by Terminal dUTP Nick-End Labeling (TUNEL) assay using the In-Situ Cell Death Detection Kit, Fluorescein (11684795910, Roche, Diagnostics, Mannheim, Germany), following the manufacturer’s instructions. As a positive control, tissue sections of wild-type zebrafish were incubated for two min with permeation solution (0.1% Tritón-X100, 0.1% sodium citrate) followed by incubation with DNase I solution (3 U/mL DNase, 50 mM Tris-HCl pH 7.5, 1 mg/mL FBS) for 10 min. DNase I treatment was omitted in the negative controls. Samples were stained with DAPI for nuclear staining, mounted in DAKO Fluorescent Mounting Medium and examined under an Eclipse Ti microscope (Nikon, Tokyo, Japan). For both GGAP immunodetection and TUNEL, three random fields per tissue section were examined by a single masked observer. Two tissue sections/eye were employed for each technique.

### 2.9. CRISPR/Cas9 Gene Editing

Target selection and sgRNA design were performed using the Custom Alt-R CRISPR-Cas9 guide RNA (https://eu.idtdna.com/site/order/designtool/index/CRISPR_CUSTOM) and CHOPCHOP V.3 programs (http://chopchop.cbu.uib.no). Potential off-target sites and highest on-target activity of sgRNAs common to both programs were assessed with CRISPR-Cas9 guide RNA design checker (https://eu.idtdna.com/site/order/designtool/index/CRISPR_SEQUENCE). To disrupt the *guca1c* gene, a pair of crRNAs (36 ng/µL each) targeting exon 1 (*guca1c*E1g1, 5′-GGAGATGCA GGGCATGACGG-3′ and *guca1c*E1g2, 5′-GGAGGAGGCCAGCAGCTACG-3′) and tracrRNA (67 ng/µL) were mixed, incubated for 5 min at 95 °C and cooled at room temperature. The crRNAs/tracrRNA complexes were mixed with the Cas9 protein (250 ng/µL) and incubated for 10 min at 37 °C to form the ribonucleoprotein (RNP) complex. The RNP complexes were injected (3 nL) into the animal pole of one-cell stage embryos (50–250 embryos/experiment) using a Femtojet 5247 microinjector (Eppendorf, Hamburg, Germany) under a Nikon DS-Ri2 stereomicroscope. All reagents for *CRISPR/Cas9* gene editing were provided by Integrated DNA Technologies, Inc. (Coralville, IA, USA).

### 2.10. Zebrafish DNA Extraction

Adult fishes were anesthetized with 0.04% tricaine methanesulfonate (MS222) and euthanized by prolonged immersion in MS222 (200–300 mg/L). Larvae were treated with 0.02% MS222. PCR-ready genomic DNA was isolated from whole zebrafish embryos (24 hpf) and from the caudal fin of anesthetized larvae (6 dpf) or adult zebrafish using the HotSHOT method [25]. Briefly, tissue samples were incubated with 20 µL of base solution (25 mM KOH, 0.2 mM EDTA) at 95 °C for 30 min in a thermal cycler C100 (BIORAD, Hercules, CA, USA), then 20 µL of neutralization buffer (40 mM TrisHCl, pH 5) were added.

### 2.11. Genotyping by High-Resolution Melting (HRM)

For HRM genotyping the PCR reactions (10 μL total volume) were prepared using 5 μL of MeldtDoctor HRM Master Mix (#4415440, Thermo Fisher Scientific), 1.5 μL (3 μM) of each primer (*guca1c*HRMFw, 5′-GTCAGGCTTGATTAGCGTGTTC-3′; *guca1c*HRMRv, 5′-AAACTCATTTATTGC GCGTGTT-3′) and genomic DNA (2 μL). The PCR was performed in a 7500 Fast real-time PCR system thermal cycler (Thermo-Fisher Scientific). Thermocycling included an initial denaturation step at 95 °C for 10 min, followed by 40 cycles consisting of 15 s denaturation at 95 °C and a combined annealing and extension step at 60 °C for 60 s. The melt curve stage consisted of the following steps: denaturation at 95 °C for 10 s, annealing at 60 °C for 60 s, HRM at 95 °C for 15 s and a final step of annealing at 60 °C for 15 s. The raw melting curve data were processed by the High-Resolution Melt Software v. 3.0.1 (Thermo-Fisher Scientific).

### 2.12. Characterization of the CRISPR/Cas9-Induced Mutation

To characterize the KO mutation, DNA samples were analyzed by Sanger sequencing using the following primers: *guca1c*SeqFw, 5′-GCAGCGGCAGACTCTTCACATCTCG-3′; *guca1c*SeqRv, 5′-TAGTGGAGCTCTAAAACCTGAAATGAATGGG-3′.

### 2.13. Statistics

Statistical comparisons between groups were performed using either the *t*-test or the one-way ANOVA using the SigmaStat 2.0 software (Systat Software Inc., San Jose, CA, USA).

## 3. Results

### 3.1. Identification of Rare GUCA1C Variants by WES

**Figure 1:**
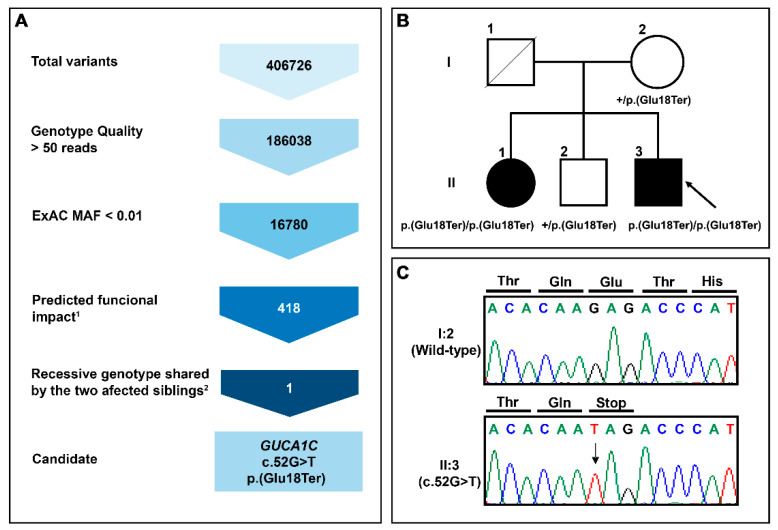
(**A**) Variant filtering scheme used for candidate variant identification in family PCG-94. Gene variants were identified by whole-exome sequencing and filtered as indicated to the left to identify candidate variants. ^1^Frameshift, stop gained, start lost, splicing acceptor, and missense variant. ^2^Homozygous or compound heterozygous genotype in the two affected siblings; (**B**) Pedigree analysis of *GUCA1C* variants in a patient with primary congenital glaucoma. The black symbol indicates the presence of the disease. The arrow in the pedigree shows the index case. +: Wild-type allele; (**C**) Confirmation by Sanger sequencing of the variant identified in this family.

This study is an extension of our previous WES analysis of 26 severe PCG cases diagnosed before the fourth month of life, that were ruled out as carrying *CYP1B1* alterations, and for which we reported no shared disease-causing genetic alterations [14]. Herein, we focus on the identification of genetic alterations underlying PCG in a family with two affected siblings (PCG-94). To that end, we designed a variant filtering algorithm aimed at identifying rare coding variants with predicted moderate or high functional impact (frameshift, nonsense, missense, and donor/acceptor splicing sites) with recessive inheritance (variants in homozygosis or compound heterozygosis) and shared by the two PCG siblings. This filtering pipeline identified only one homozygous nucleotide substitution in the *GUCA1C* gene (c.52G > T), predicted to result in a nonsense variant (p.(Glu18Ter), Figure 1A) that likely leads to the complete LoF of the gene product. This variant is reported in the gnomAD v2.1.1 database with low frequency (0.004513) and with three homozygous genotypes. The segregation of the variant was confirmed by Sanger sequencing (Figure 1B,C).

### 3.2. Clinical Features of Patients with the Nonsense GUCA1C Variant

The proband was diagnosed with bilateral congenital glaucoma at birth in a different Hospital and currently he is 48 years old. He attended the Ophthalmology Department of San Carlos Hospital with endophthalmitis at the age of 28 years. The patient presents with amaurosis in the right eye and light perception in the left eye (LE), bilateral severe optic nerve excavation and an IOP value of 17 mmHg in the LE with (Table 1). The patient was subjected to several bilateral glaucoma operations and requires two drugs (brimonidine tartrate and timolol) to control the IOP in the LE. Ocular examination also revealed bilateral megalocornea, Haab’s striae and leukoma, as well as band keratopathy in the left eye (Table 1). Additional ophthalmological features are presented in Table 1. 

The patient’s siter was 36 years old at the time of the study and was diagnosed with unilateral congenital glaucoma in a different hospital. Additional clinical information was not available.

### 3.3. Expression of GCAP3 in Adult Human Ocular Tissues

**Figure 2:**
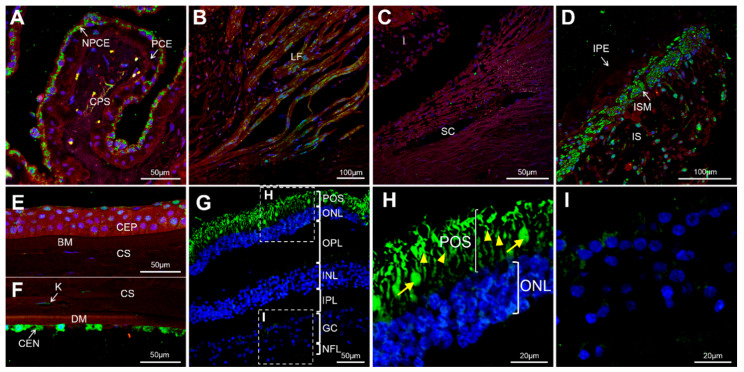
Detection of GCAP3 in human ocular tissues by confocal fluorescence immunohistochemistry; histological sections (10 µm) of a human eye from a 45-year-old Caucasian female donor were incubated with rabbit anti-human GUCA1C primary antibody (1:200) and donkey Cy2-anti-rabbit secondary antibody (1:1000); (**A**) Confocal wide-field micrographs of ciliary processes; (**B**) Ciliary muscle; (**C**) Trabecular meshwork; (**D**) Iris; (**E**) Corneal epithelium; (**F**) Corneal endothelium and (**G**) Retina. (**H**,**I**) detailed images of photoreceptors and the ganglion cell layer, respectively. Both rods (yellow arrowheads) and cones (yellow arrows) showed positive GCAP3 immunoreactivity (**H**). Green, blue and red signals correspond to GCAP3 immunoreactivity, DAPI nuclear staining and tissue autofluorescence, respectively. BM: Bowman’s membrane. CEN: Corneal endothelium. CEP: Corneal Epithelium. CS: Corneal stroma. CPS: Ciliary process stroma. DM: Descemet’s membrane. GC: Ganglion cells. I: Iris. INL: Inner nuclear layer. IPL: Inner plexiform layer. IPE: Iris pigmented epithelium. IS: Iris stroma. ISM: Iris sphincter muscle. K: Keratocyte. LF: Longitudinal fibers. NFL: Nerve fiber layer. NPCE: Non-pigmented ciliary epithelium. ONL: Outer nuclear layer. OPL: Outer plexiform layer. PCE: Pigmented ciliary epithelium. POS: Photoreceptor outer segments. SC: Schlemm’s canal. The negative controls are shown in Figure S1.

To the best of our knowledge the presence of the GCAP3 protein in adult human ocular anterior segment tissues has not been studied. FIHC using a commercial anti-human GUCA1C antibody showed labeling of the ciliary body with intense cytoplasmatic immunoreactivity in the non-pigmented ciliary epithelium (NPCE) and immunostaining of ciliary muscle longitudinal fibers (Figure 2A,B). No positive immunolabeling was detected in the trabecular meshwork (Figure 2C). The iris sphincter muscle was highly positive for GCAP3 (Figure 2D). Some iris stroma cells, probably fibroblasts, also showed the presence of immunoreactivity for this protein (Figure 2D). The anti-GUCA1C antibody also labeled cells of the corneal epithelium, keratocytes, and especially the corneal endothelium (Figure 2E,F). As expected, the retina was also positive for GCAP3, photoreceptors (both rods and cones) were immunoreactive against GCAP3 with strong signals in the inner and outer segments (Figure 2G,H). Some cells in the retinal ganglion layer showed a diffuse immunostaining (Figure 2I). The specificity of these signals was supported by their absence in the corresponding negative controls (Figure S1). These results clearly show the expression of GCAP3 in the tissues of the ocular anterior segment involved in the production and exit of the aqueous humor, suggesting, along with previous studies [26,27], that this protein plays a role in IOP homeostasis and that its functional disruption may contribute to glaucoma.

### 3.4. Functional Analysis of guca1c in Zebrafish

**Figure 3:**
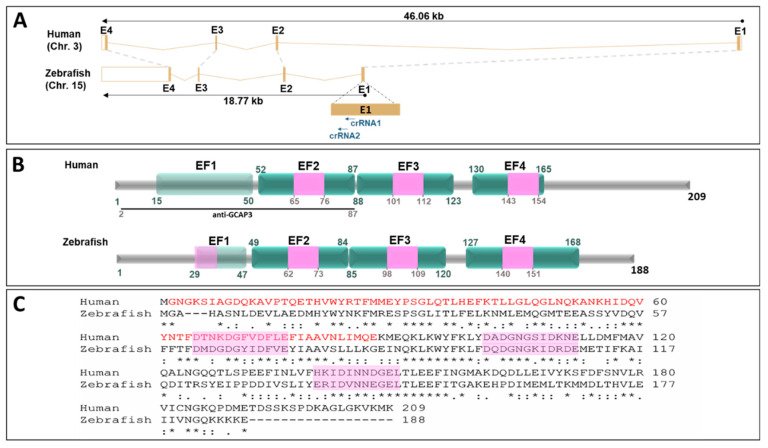
Structural conservation of human *GUCA1C* and its ortholog zebrafish gene and protein. (**A**) Genomic alignment of human *GUCA1C* (ENSG00000138472) and zebrafish *guca1c* (ENSDARG00000030758) genes. The dotted gray lines show the correspondence of conserved exons. The black arrows indicate the sense of transcription. The blue arrows indicate the localization of the crRNAs used in this study. The “Ensembl region comparison tool” was used to obtain this image. E: Exon; (**B**) Human and zebrafish GCAP3 protein domain conservation (ENST00000261047.8 and ENSDART00000043226.7, respectively). Domains are indicated according to the Prosite database (https://prosite.expasy.org/). The numbers correspond to amino acid positions. EF: EF-hand calcium-binding domain. Pink boxes: Calcium-binding sites. Nonfunctional EF-hands are indicated by light green boxes. Black line: Peptide used as an antigen to obtain the commercial anti-CGAP3 antibody used in this study; (**C**) Amino acid sequence alignment of human and zebrafish GCAP3 proteins. The alignment was carried out with ClustalW14 (https://www.ebi.ac.uk/Tools/msa/clustalo/) [31]. The asterisks indicate the positions where all the amino acids are identical. Two vertical dots show amino acids with similar chemical properties. One dot denotes amino acid positions with weak chemical similarity. Pink boxes: Calcium-binding sites.

To further evaluate the role of *GUCA1C* LoF in congenital glaucoma and retinal physiology, we used zebrafish as an animal model. The human *GUCA1C* gene is composed of four exons and is located on chromosome 3. Two orthologous genes—*guca1c* and *guca1d*—have been identified in the zebrafish genome on chromosomes 15 and 21, respectively. Both genes emerged via a gene duplication event from an ancestral gene common to other teleost fishes [28]. Moreover, the expression pattern for both *guca1c* and *guca1d* genes in zebrafish larvae is described as being very similar [29,30]. Because there is no evidence of functional divergence among these genes, we prioritized *guca1c* LoF analysis in our study, although possible compensatory phenotypic effects by *guca1d* on a *guca1c* KO background cannot be disregarded. DNA sequence comparison analyses of the human and zebrafish GUCA1C genes show similar overall intron-exon organization (Figure 3A) and the corresponding GCAP3 proteins present conserved EF-hand calcium-binding domains (Figure 3B) and 44% amino acid identity (Figure 3C).

**Figure 4:**
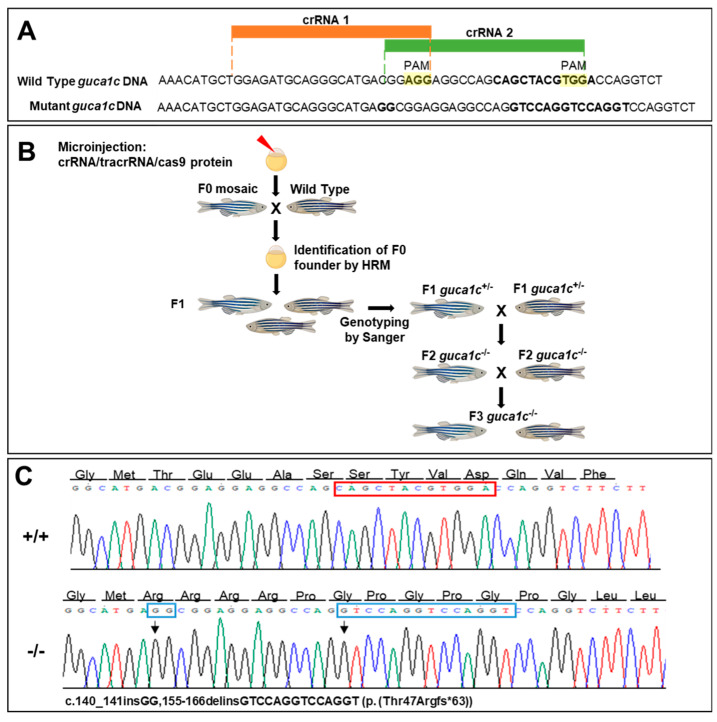
Generation of a *guca1c* KO zebrafish line by CRISPR/cas9 genome editing. (**A**) Localization of the exon 1 target sequences selected to design the two crRNAs. The PAM sites are highlighted in yellow. Two simultaneous crRNAs were used to ensure gene disruption and to facilitate identification of F0 mutants by HRM; (**B**) Stepwise procedure followed to establish the KO line described in this study. Successfully mutagenized F0 animals were raised to adulthood and crossed with wild-type partners. F1 animals were genotyped by Sanger sequencing and crossed with wild-type subjects to segregate off-targets in the F2. (**C**) Electropherograms of the mutations obtained. The arrows indicate the location of the mutations. The red and blue rectangles indicate deleted and inserted nucleotides, respectively. The cartoon in panel (**B**) was created with BioRender.com.

To ensure disruption of the zebrafish *guca1c* gene and to facilitate the identification of F0 mutants by HRM, we used simultaneously two overlapping crRNAs targeting exon1 (Figure 3A and Figure 4A). The RNP complexes (crRNAs/tracrRNA and Cas9 protein) were co-injected into the animal pole of AB zebrafish at the one-cell stage of development (Figure 4B). The injected embryos (F0) were raised to adulthood and screened by HRM for the presence of germ-line transmitted *guca1c* mutations (Figure 4B). F0 mutant mosaic fishes were outcrossed with wild-type animals and the offspring (F1) was screened for *guca1c* mutations by HRM. Several fishes were identified as transmitting mutations; Sanger sequencing identified one animal harboring an indel variant (c. 140_141insGG,155-166delinsGTCCAGGTCCAGGT, NM_194393) (Figure 4C). It was predicted that this mutation would result in a frameshift and a premature termination codon (PTC) in the new open reading frame (p.(Thr47Argfs*63, NP_919374)) (Figure 4C). This individual was selected to establish the null *guca1c* zebrafish line. Mutant F1 heterozygotes were backcrossed with wild-type AB zebrafish to produce the F2 offspring. The F3 progeny was obtained by inbreeding F2 heterozygotes (Figure 4B); homozygous mutant *guca1c* fishes were identified by Sanger sequencing. 

**Figure 5:**
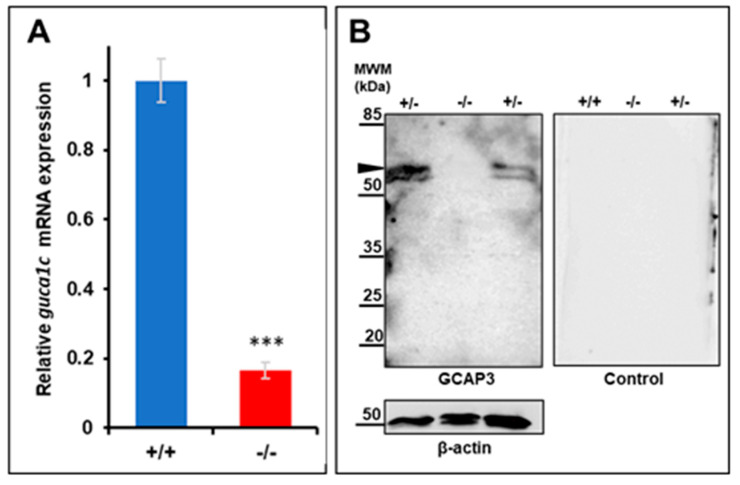
Molecular characterization of the *guca1c* KO zebrafish line. (**A**) qPCR of *guca1c* mRNA levels in pools of 50 zebrafish larvae (6 dpf). The results are expressed as relative expression levels normalized to wild-type. Asterisks indicate statistical significance compared to the control: *p* < 0.001 (***); (**B**) Western immunoblot analysis of GCAP3 in zebrafish larvae (6 dpf). Western blotting was carried out with protein extracts (75 μg total protein) obtained from pools of eight larvae (6 dpf). GCAP3 was detected using an anti-GUCA1C primary antibody (1:50) (left panel). As a loading control, the membrane was stripped and incubated with an anti-β-actin antibody (1:500) (bottom panel); a replica of the membrane was analyzed in parallel with the secondary goat anti-rabbit HRP antibody (1:1000) as a negative control (right panel). The arrowhead indicates a positive double band over 50 KDa, which may correspond to a GCAP3 dimer. MWM: Molecular weight marker. +/+: Wild-type; +/-: KO heterozygote; -/-: KO homozygote.

The selected mutation is expected to lead to NMD-dependent mRNA degradation, resulting in LoF of the *guca1c* gene and the absence of the GCAP3 zebrafish protein activity. To confirm this idea, *guca1c* expression was analyzed by qRT-PCR. We observed that *guca1c* mRNA levels in homozygous mutant larvae (6 dpf) were less than 40% of the value determined in their wild-type littermates (Figure 5A), in accordance with our hypothesis. On the other hand, if the residual mutant mRNA was translated, it would result in a truncated and likely, null protein because it would lack most of the functional domains of the normal protein. Western blot analysis of GCAP3 in wild-type zebrafish larvae (6 dpf) using the same commercial antibody as that used for the immunohistochemistry of human tissues showed a specific doublet higher than 45 kDa (Figure 5B), which, in accordance with previous reports, may correspond to a dimer of the protein [32]. As expected, the intensity of this signal in homozygous mutant larvae was undetectable and reduced in heterozygous larvae. Parallel control western blot analyses of beta-actin showed no significant differences in sample loading. Altogether, these results support a *guca1c* LoF in the generated zebrafish line.

### 3.5. Phenotypic Characterization of guca1c KO Zebrafish

**Figure 6:**
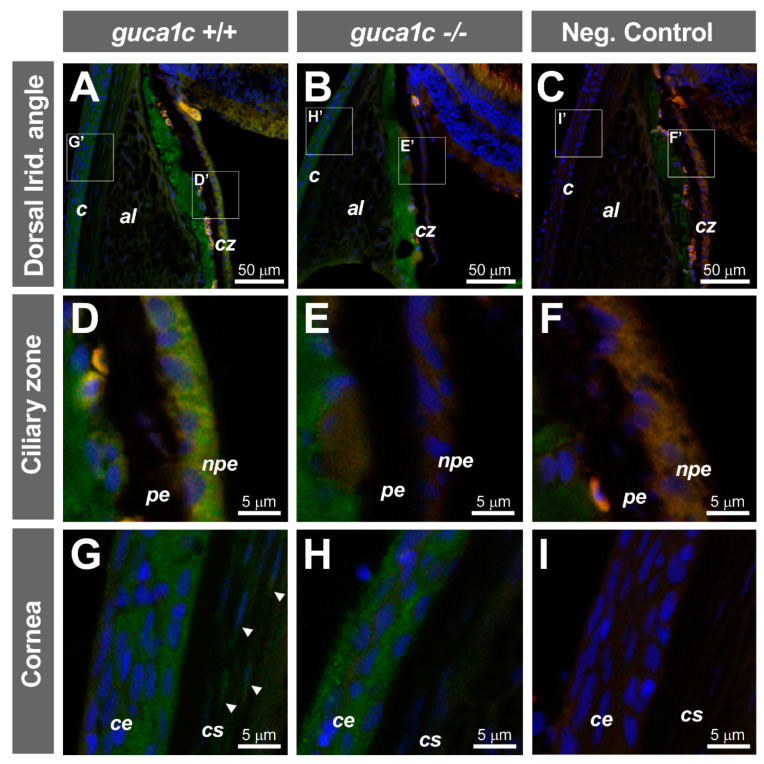
Localization of GCAP3 in ocular anterior segment tissues of adult zebrafish (6 months) by confocal fluorescence immunohistochemistry. (**A**,**B**) Histological sections (10 µm) of wild-type (+/+) or *guca1c* KO (-/-) adult zebrafish eyes were incubated with either a rabbit anti-GUCA1C primary antibody and a donkey Cy2-anti-rabbit secondary antibody or (**C**) with only the secondary antibody as a negative control; (**D**–**I**) Confocal wide-field micrographs of panels correspond to digital magnifications of insets (D’-I’) indicated in panels (**A**–**C**). Green, blue and red signals correspond to GCAP3 immunoreactivity, DAPI nuclear staining, and tissue autofluorescence, respectively. Arrowheads indicate keratocytes. The images are representative of the results observed in three fishes of each genotype. *c*: Cornea; *al*: Annular ligament; *cz*: Ciliary zone; *pe*: Ciliary pigmented epithelium; *npe*: Non-pigmented ciliary epithelium; *ce*: Corneal epithelium; *cs*: Corneal stroma.

No significant gross external macroscopic alterations were observed in either larvae (96 hpf) or adult (6 months) *guca1c* KO fishes. Immunohistochemical analysis of GCAP3 in adult (6 months) wild-type and KO zebrafish eyes revealed immunopositive signals in the non-pigmented epithelium of the ciliary zone (Figure 6A,D), which is involved in aqueous inflow. Additional GCAP3 immunoreactivity was observed in the corneal epithelium (Figure 6G) and weak signals were observed in keratocytes (Figure 6G, arrows). The GCAP3 immunoreactivity in the non-pigmented epithelium and keratocytes was absent in the KO animals (Figure 6B,E,H). Nevertheless, the positive labeling remained in the corneal epithelium of KO zebrafishes, indicating a possible antibody cross-reaction with other GCAPS (Figure 6H). The absence of immunoreactivity in the negative controls indicated the specificity of the primary antibody (Figure 6C,F,I). 

**Figure 7:**
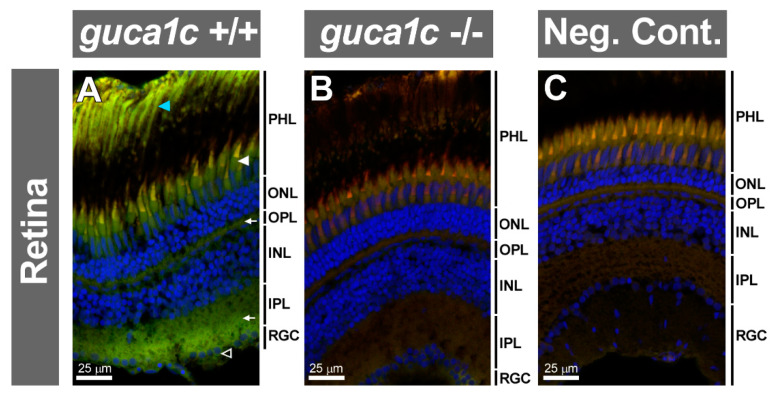
Detection of GCAP3 in the adult zebrafish eye retina (6 months) by confocal fluorescence immunohistochemistry. (**A**,**B**) Histological sections (10 µm) of wild-type (+/+) or *guca1c* KO (-/-) adult zebrafish eyes were incubated with either a rabbit anti-GUCA1C primary antibody and a donkey Cy2-anti-rabbit secondary antibody; (**C**)The primary antibody was omitted in the negative control. Green, blue, and red signals correspond to GCAP3 immunoreactivity, DAPI nuclear staining, and tissue autofluorescence, respectively. Specific labeling in photoreceptors, rods and cones (blue and white arrowheads, respectively), outer and inner plexiform layers (arrows), and ganglion cells (empty arrowhead) are indicated. PHL: Photoreceptor layer. ONL: Outer nuclear layer. OPL: Outer plexiform layer. INL: Inner nuclear layer. IPL: Inner plexiform layer. RGC: Retinal ganglion cells. The images are representative of the results observed in three fishes of each genotype.

Immunohistochemistry of the wild-type retina detected GCAP3 immunoreactivity in photoreceptors (rods and cones) (Figure 7A, blue and white arrowheads, respectively), inner and outer plexiform layers (Figure 7A, arrows) and ganglion cell layer (Figure 7A, empty arrow). These immunosignals were absent in the KO retina and the negative control, demonstrating their specificity (Figure 7B,C, respectively). No gross structural alterations were observed in the retina of KO zebrafish using this technique.

In accordance with the previous result, histological analysis of toluidine-blue-stained tissue sections did not revealed any gross structural retinal alteration in the KO zebrafish (Figure S2A,B), however, it did show thinning of the KO corneal epithelium (5.1 ± 0.2 µm) compared to that of wild-type animals (8.6 ± 0.2 µm) (Figure S2C,D).

**Figure 8:**
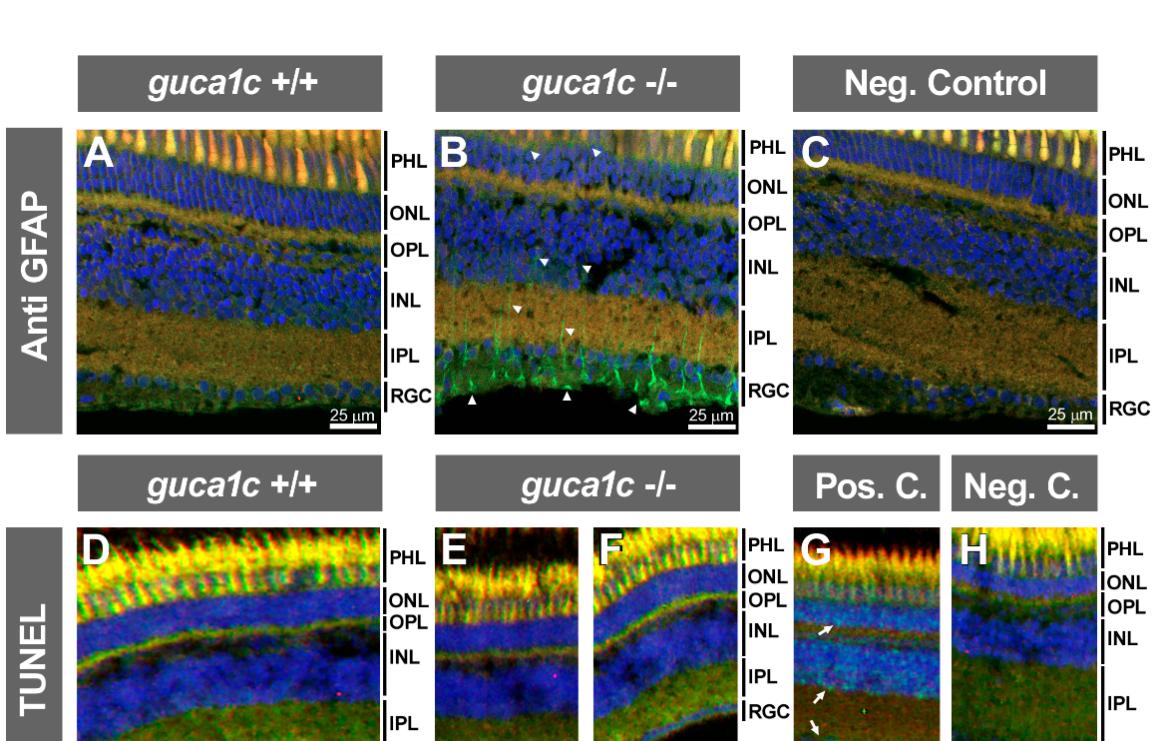
Retinal gliosis and ganglion cell apoptosis in adult (6 months) *guca1c* KO zebrafish. (**A**,**B**) Immunolabeling against GFAP (green) was used to analyse glial activation in histological sections (10 µm) of zebrafish eyes incubated with either a rabbit anti-GFAP primary antibody (1:50) and a donkey Cy2-anti-rabbit secondary antibody (1:1000). GFAP labelling of Müller cells (green signal) was absent in wild-type zebrafish (**A**) and present in the KO animals (**B**, arrowheads), indicating retinal gliosis; (**C**) The primary antibody was omitted in the negative control; (**D**,**H**) Apoptosis was assessed using terminal dUTP nick-end labeling (TUNEL) of fragmented DNA; (**D**) TUNEL-positive cells were not detected in the retina of wild-type zebrafish; (**E** and **F**) TUNEL-positive retinal ganglion cells were observed in two independent microscopic fields of the KO zebrafish (arrows); (**G**,**H**) Retinal sections of adult zebra fish incubated with DNase I or with only the labeling solution were used as a positive and negative controls, respectively. Note the presence of positive cells in different retinal layers (arrows); Microphotographs in panels (**D**–**H**) were obtained using a Nikon Eclipse Ti microscope. Blue and red signals correspond to DAPI nuclear staining and tissue autofluorescence, respectively. INL: Inner nuclear layer. IPL: Inner plexiform layer. ONL: Outer nuclear layer. OPL: Outer plexiform layer. PHL: Photoreceptor layer. RGC: Retinal ganglion cells. +/+: wild-type; -/-: *guca1c* KO. The images are representative of the results observed in two fishes of each genotype. Two tissue sections per eye were analyzed.

Next, we evaluated retinal injury in the KO animals by immunohistochemistry. To that end, we selected two wild type and two KO zebrafish. First, we used an antibody raised against the glial fibrillary acidic protein (GFAP). No detectable immunoreactivity was observed in the retina of wild-type animals (Figure 8A); however, a significant upregulation of this protein was present in Müller cells of KO retinas, indicating the existence of gliosis associated to GCAP3 deficiency (Figure 8B, arrowheads). GFAP immunoreactive cells were observed bilaterally in the two fishes, with an average number of 14 positive cells per microscopic field in the ganglion cell layer. The absence of immunoreactivity in the negative control, carried out with only the secondary antibody, indicated the specificity of the primary antibody (Figure 8C). In addition, analysis of retinal apoptosis by TUNEL assay showed the absence of labeling in the retina of wild-type animals (Figure 8D) while revealed some positive nuclear signals in the ganglion cell layer of KO animals (Figure 8E,F, arrows). Apoptotic cells were detected bilaterally in one KO fish and unilaterally in the second animal analyzed (three positive cells were observed in total). The presence of nuclear staining in different retinal layers (particularly ganglion and outer nuclear cells) of the positive control (Figure 8G, arrows), along with the absence of positive cells in the negative control (Figure 8H), supported the specificity of the signals.

## 4. Discussion

In this study we have identified *GUCA1C* LoF as the likely genetic alteration underlying PCG in only one out of 26 families of our cohort. Given the absence of the disease in previous generations of the family, the presence of an affected brother/sister pair, and the presence of one non-affected sibling, we assumed a recessive disease inheritance as the most probable cause of the disease. In addition, these features made unlikely the existence of disease-associated de novo variants. Whole-exome sequencing revealed the presence of one homozygous nonsense *GUCA1C* variant in the two affected siblings that segregated with the disease following the expected autosomal recessive inheritance. We also applied filters to identity rare compound heterozygous variants carried by the two affected siblings, with a high and moderate predicted functional effect, but we did not identify any variant of this type. The *GUANYLATE CYCLASE ACTIVATOR 1C* (*GUCA1C*) gene encodes the guanylate cyclase 3 activating protein (GCAP3), a calcium-binding protein that belongs to the calmodulin gene superfamily [33]. This protein is composed of 209 amino acids (23.8 kD) and presents 57% and 49% amino acid similarity with GCAP1 and GCAP2, respectively. Like other GUCAs, GCAP3 contains a putative N-myristoylation site at Gly 2 and three putative EF-hand motifs involved in Ca(2+) binding [33]. Guanylate cyclase-activating proteins are present in human photoreceptors and retinal ganglion cells [34], and in general, act as regulators of photoreceptor guanylate cyclases playing a role in phototransduction [35]. It has also been found that guanylate cyclase activators increase the rate of aqueous humor outflow leading to IOP reduction [26] through cGMP-dependent regulation of Schlemm’s canal cell volume and TM cell volume [27]. Because of this property, guanylate cyclase activators have been proposed as therapeutic agents for glaucoma [26,27]. In addition, it has been reported that a KO mouse for the α-1 subunit of soluble guanylate cyclase developed glaucoma [36]. Based on these data, we hypothesize that the loss of *GUCA1C* function could result in decreased cGMP, increased IOP, and glaucoma. In accordance with these ideas, the nonsense homozygous *GUCA1C* variant (p.(Glu18Ter)) identified in the two siblings affected with PCG, may disrupt cGMP synthesis, resulting in IOP elevation and glaucoma. To the best of our knowledge, no genetic *GUCA1C*-associated disease has been identified; however, the alteration of other related genes, such as *GUCA1A*, produces dominant cone or rod and cone dystrophies due to gain-of-function alleles that result in a constitutively active protein [37,38]. The pathogenicity of the nucleotide substitution identified in this family (c.52G > T) is supported by the predicted premature termination codon (PTC) generated in the so-called “NMD boundary” of the mutant mRNA, located 50–55 bases 5′ of the last exon-exon junction [39]. This likely leads to the complete LoF of the mutant *GUCA1C* gene product through mRNA degradation by nonsense-mediated decay (NMD) [40]. PTCs are often associated with severe phenotypes, and PTC-associated diseases account for approximately 11% of all genetic lesions causing genetic diseases [41]. The overall reported frequency of this variant in gnomAD v2.1.1 is below 0.5% with three homozygous genotypes, two of them correspond to cases while the third one corresponds to one control. This finding may indicate the existence of incomplete penetrance in these subjects due to functional compensation by paralogous genes such as *GUCA1A*, *GUCA1B*, or other functionally related genes. Additionally, very limited phenotypic information is available for these subjects; therefore, we cannot completely rule out the idea that at least some of them present with glaucoma. Identification of *GUCA1C* LoF in only one out of 26 PCG cases indicates that this gene may contribute to PCG in a small proportion of cases, which provides further support for the notion of elevated genetic heterogeneity in this disease. Alternatively, and although we believe it highly unlikely, the possibility that this variant is unrelated to the disease cannot be completely ruled out. Thus, further studies are required to firmly establish its pathogenicity. The presence of GCAP3 in glaucoma-related human ocular tissues also supports the idea that functional disruption of *GUCA1C* plays a role in this disease. In fact, to the best of our knowledge, we report for the first time the presence of this protein in aqueous humor inflow tissues, suggesting that functional alteration of *GUCA1C* may disrupt the balance between AH production and drainage, resulting in elevated IOP and glaucoma. In adult retinal tissue, GCAP3 was detected both in rod and cone photoreceptors according to its role in phototransduction. Previous studies have detected GCPA3 only in cone photoreceptor cells, probably due to limitations of the technique [32]. In addition, our results showed GCAP3 immunoreactivity in the ganglion cell layer. In line with this data, GCAP2 immnuoreactivity has also been reported in the human retina [34]. Overall, these data suggest that functional disruption of *GUCA1C* might also cause direct retinal damage, but this hypothesis could not be clinically evaluated in our study because the advanced disease in the patient who carried the LoF variant in this gene.

In an additional effort to evaluate the pathogenicity of GUCA1C LoF in glaucoma and retinal function, we analyzed the expression of this protein in the zebrafish eye and generated a *guca1c* KO zebrafish line using CRIPS/Cas9 genome editing. In accordance with the expression in human ocular tissues, GCAP3 was detected in aqueous humor inflow tissues (i.e., the non-pigmented epithelium of the ciliary zone), photoreceptors and ganglion cells. In line with these results, it has been reported that the gene is expressed mainly in human and zebrafish retinal cones [32]. qRT-PCR, Western blot, and immunohistochemical analyses supported the LoF in the zebrafish line, though, no gross macroscopic alterations were observed in adult KO animals. Histology analyses revealed thinning of the corneal epithelium with an apparently unaltered corneal stroma, indicating that *guca1c* is required for the normal structure of the cornea. One of the most interesting phenotypic findings in *guca1c* KO animals was the upregulation of GFAP in Müller cells, and the evidence of apoptosis in some ganglion, indicating the existence of gliosis and glaucoma-like alterations associated with GCAP3 LoF. Increase of the GFAP intermediate filament protein is a universal early cellular marker for retinal injury [42]. Although it is difficult to determine the cause of Müller cell activation in these knockout animals, we can speculate that it might be a cellular response triggered by deficient expression of GCAP3 in the retina. We could not assess whether retinal ganglion cell death was secondary to IOP elevation or direct ganglion cell damage; and we only analyzed ganglion cell apoptosis in adult animals, therefore, we could not determine the onset time and progression of these alterations. Moreover, the low number of TUNEL positive cells observed may indicate that ganglion cell apoptosis was not generalized in the retina at the time of the study. Additional biochemical, histological and time course analysis are required to elicit the cause, onset time and extension of ganglion cell apoptosis and retinal damage. We are aware that the paralog gene *guca1d*, and/or other functionally related genes, might compensate, at least partially, *guca1c* LoF. Therefore, further studies are required to elucidate the pathogenic and phenotypic effect of *guca1c* disruption.

## 5. Conclusions

Our results provide evidence for the role of *GUCA1C* as a novel candidate gene in PCG and offer new insights into the role that this gene plays in the anterior segment and the retina.

## Figures and Tables

**Table 1:** Ophthalmological features of the index case with *GUCA1C* variants (PCG-94).

Patient	II-3
Age at diagnosis/age at last ophthalmic revision	Birth/48 years
IOP at diagnosis (mm Hg) (RE/LE)	NA
Last IOP (mm Hg) (RE/LE)	NA/17
Last cup/disc ratio (RE/LE)	0.9-1/0.9-1
Number and type of glaucoma surgery (RE/LE)	6 (3G, 3T)/5 (3G, 2T)
Number of antiglaucoma drugs (RE/LE)	0/2, brimonidine tartrate and timolol
Visual acuity (RE/LE)	Amaurosis/Light perception
Lens	Cataract (B)
Central corneal thickness (m) (RE/LE)	740/740
Corneal morphology	MC (B), HS (B), L (B), BK and SE (RE)

B: bilateral; BK: band keratopathy; G: goniotomy; HS: Haab’s striae; IOP: intraocular pressure; LE: left eye; MC: megalocornea; NA: not available; RE: right eye; L: leukoma; SE: stromal edema; T: trabeculectomy.

## Supplementary Materials

The following are available online at https://www.mdpi.com/2073-4425/11/5/550/s1, Figure S1: Negative controls used for the fluorescence immunohistochemistry detection of GCAP3 in human ocular tissues shown in Figure 2. Figure S2: Retinal and corneal histology of adult (2 years) *guca1c* KO zebrafish. Tissue sections (10 µm) of wild-type (+/+) or *guca1c* KO (-/-) zebrafish eyes were stained with toluidine blue; (**A**) and (**B**) Retina sections of wild-type and KO animals, respectively; (**C**) and (**D**) Cornea sections of wild-type and KO animals, respectively. CE: Corneal epithelium.
