# Supplementary material for: Role of GUCA1C in Primary Congenital Glaucoma and in the Retina: Functional Evaluation in Zebrafish
Source: Genes (Basel). 2020 May 14;11(5):550. doi: 10.3390/genes11050550 (PMC7288452; doi:10.3390/genes11050550)
Supplement: Supplementary file 1 [file genes-11-00550-s001.pdf]

Supplemental Material

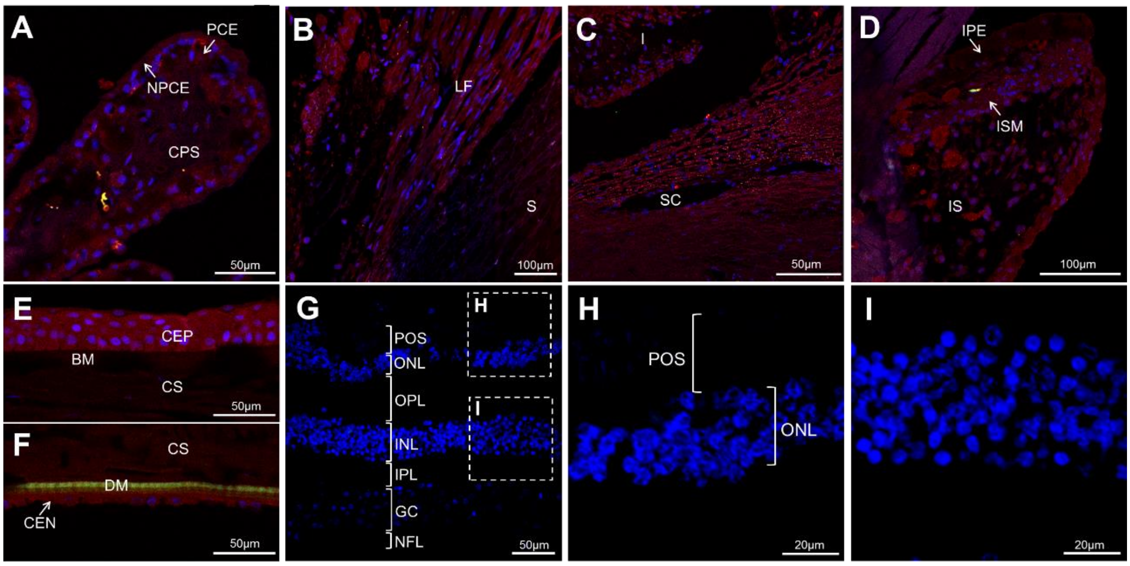

Figure S1. Negative controls used for the fluorescence immunohistochemistry detection of GCAP3 in human ocular tissues shown in Figure 2. Histological sections (10 μm) of a human eye from a 45-year-old Caucasian female donor were incubated with only a donkey Cy2-anti-rabbit secondary antibody (1:1000); (**A**) Confocal wide-field micrographs of ciliary processes; (**B**) Ciliary muscle; (**C**) Trabecular meshwork; (**D**) Iris; (**E**) Corneal epithelium; (**F**) Corneal endothelium and (**G**) Retina; (**H** and **I**) detailed images of photoreceptors and the ganglion cell layer, respectively. Blue and red signals correspond to DAPI nuclear staining and tissue autofluorescence, respectively. Note the absence of green fluorescence corresponding to the secondary antibody. BM: Bowman's membrane. CEN: Corneal endothelium. CEP: Corneal Epithelium. CS: Corneal stroma. CPS: Ciliary process stroma. DM: Descemet's membrane. GC: Ganglion cells. I: Iris. INL: Inner nuclear layer. IPL: Inner plexiform layer. IPE: Iris pigmented epithelium. IS: Iris stroma. ISM: Iris sphincter muscle. LF: Longitudinal fibers. NFL: Nerve fiber layer. NPCE: Non-pigmented ciliary epithelium. ONL: Outer nuclear layer. OPL: Outer plexiform layer. PCE: Pigmented ciliary epithelium. POS: Photoreceptor outer segments. SC: Schlemm's canal.

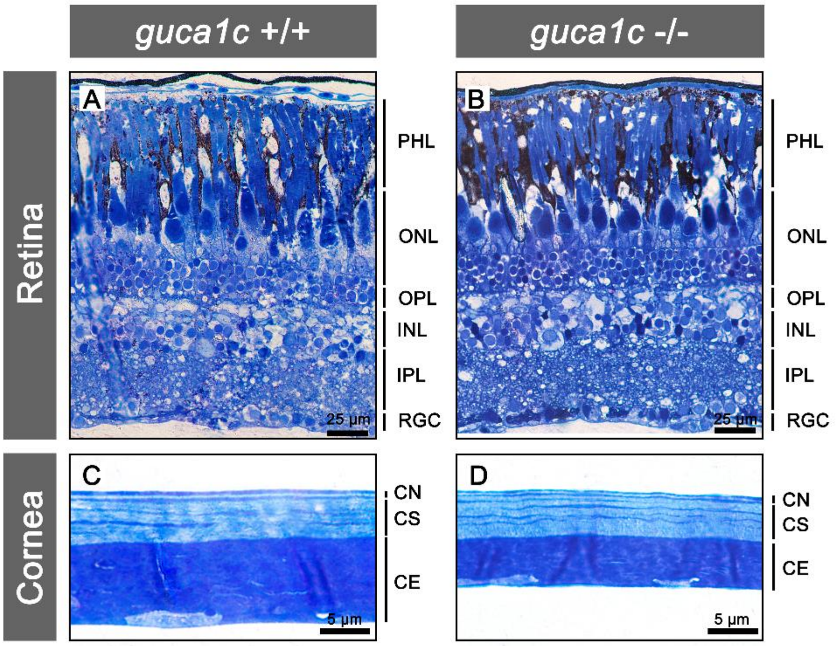

Figure S2. Retinal and corneal histology of adult (2 years) *guca1c* KO zebrafish. Tissue sections (10 µm) of wild-type (+/+) or *guca1c* KO (-/-) zebrafish eyes were stained with toluidine blue; (**A**) and (**B**) Retina sections of wild-type and KO animals, respectively; (**C**) and (**D**) Cornea sections of wild-type and KO animals, respectively. CE: Corneal epithelium. CN: Corneal endothelium. CS: Corneal stroma. INL: Inner nuclear layer. IPL: Inner plexiform layer. ONL: Outer nuclear layer. OPL: Outer plexiform layer. PHL: Photoreceptor layer. RGC: Retinal ganglion cells. The images are representative of the results observed in two fishes of each genotype.
